# Anti-Inflammatory Nutrients and Obesity-Associated Metabolic-Inflammation: State of the Art and Future Direction

**DOI:** 10.3390/nu14061137

**Published:** 2022-03-08

**Authors:** Giuseppe Grosso, Daniela Laudisio, Evelyn Frias-Toral, Luigi Barrea, Giovanna Muscogiuri, Silvia Savastano, Annamaria Colao

**Affiliations:** 1 Department of Biomedical and Biotechnological Sciences, University of Catania, 95123 Catania, Italy; giuseppe.grosso@unict.it; 2 Dipartimento di Medicina Clinica e Chirurgia, Sezione di Endocrinologia, Università “Federico II” di Napoli, Via Sergio Pansini, 5, 80131 Naples, Italy; daniela.laudisio@libero.it (D.L.); sisavast@unina.it (S.S.); colao@unina.it (A.C.); 3 Centro Italiano per la cura e il Benessere del Paziente con Obesità (C.I.B.O), Dipartimento di Medicina Clinica e Chirurgia, Sezione di Endocrinologia, Università “Federico II” di Napoli, Via Sergio Pansini, 5, 80131 Naples, Italy; luigi.barrea@unina.it; 4 School of Medicine, Santiago de Guayaquil Catholic University, Av. Pdte. Carlos Julio Arosemena Tola, Guayaquil 090615, Ecuador; evelynft@gmail.com; 5 Dipartimento di Scienze Umanistiche, Università Telematica Pegaso, 80132 Napoli, Italy; 6 Cattedra Unesco “Educazione Alla Salute e Allo Sviluppo Sostenibile”, Federico II University, 80131 Naples, Italy

**Keywords:** diet, nutrition, inflammation, food groups, dietary patterns, macronutrients, phytochemicals, polyphenols, c-reactive protein, inflammatory biomarkers, whole grains, fiber, meat, legumes, dairy, milk, olive oil, Mediterranean diet, dash diet, vegetable, fruit, plant-based diet

## Abstract

Growing evidence supports the hypothesis that dietary factors may play a role in systemic low-grade chronic inflammation. Summary evidence from randomized controlled trials has shown substantial effects on biomarkers of inflammation following the adoption of plant-based diets (including, but not limited to, the Mediterranean diet), while consistent findings have been reported for higher intakes of whole grains, fruits, and vegetables and positive trends observed for the consumption of legumes, pulses, nuts, and olive oil. Among animal food groups, dairy products have been shown to have the best benefits on biomarkers of inflammation, while red meat and egg have been shown to have neutral effects. The present review provides an overview of the mechanisms underlying the relation between dietary factors and immune system, with a focus on specific macronutrient and non-nutrient phytochemicals (polyphenols) and low-grade inflammation. Substantial differences within each macronutrient group may explain the conflicting results obtained regarding foods high in saturated fats and carbohydrates, underlying the role of specific subtypes of molecules (i.e., short-chain fatty acids or fiber vs. long chain fatty acids or free added sugars) when exploring the relation between diet and inflammation, as well as the importance of the food matrix and the commixture of foods in the context of whole dietary patterns. Dietary polyphenols and oligopeptides have been hypothesized to exert several functions, including the regulation of the inflammatory response and effects on the immune system. Overall, evidence suggests that dietary factors may affect the immune system regardless of obesity-related inflammation.

## 1. Introduction

Over the last 100 years, nutritional sciences have attempted to understand the role of nutrition as a major determinant of health [1]. Besides the diet’s primary function of being the source of energy for human life, the science that deals with this discipline has contributed to the global knowledge of countless other functions and mechanisms affecting human health beyond calorie count. While the discoveries of the role of macro- and micronutrients have been corroborated over a wide range of time (nearly 50 years) and been well received by the general population, new and later discoveries, including the role of nutrition in low-grade inflammation, are still the focus of great attention from scientists and researchers globally [2]. As most of the research in this area is ongoing and far from conclusive, it is hard to identify definitive evidence and even harder to identify cause–effect relationships or clear pathways for isolated mechanisms, since nutrition is a complex process that cannot be considered separately from other aspects of human physiology. The involvement of the gut microbiota, the immune system, and the brain circadian cycles among the various pathways through which food affects the system’s homeostasis (as well as the other way around) provides a glimpse of how extraordinarily complex the effect of nutrition on human health is, far beyond the most obvious metabolic outcomes [3].

Among other factors, chronic subclinical low-grade inflammation has been considered to be a major risk factor for a number of conditions, including cardiovascular diseases, some neurological disorders (i.e., depression and cognitive impairment), and certain tumors (i.e., colorectal, lung, and prostate cancer, among others) [4,5,6]. Obesity has been reported to be a major determinant of low-grade chronic inflammation. As imbalance between caloric intake and energy expenditure occurs, excess energy is stored in adipocytes, which increase in number and volume: the resulting hyperplasia and hypertrophy of the adipose tissue lead to oxygen depletion and the establishment of a condition of cellular suffering that leads to the recruitment of M1 macrophages, a type of macrophage that express high levels of pro-inflammatory receptors (i.e., Toll-like receptors (TLRs), tumor necrosis factor receptors (TNFRs), and interleukin-1 receptor (IL-1R)) and high activation of the nuclear factor-kB (NF-kB) transcription factors for pro-inflammatory molecules [7]. Among the various long-term systemic effects, low-grade inflammation may affect insulin sensitivity leading to metabolism impairment and increased risk of other non-communicable diseases [8,9]. Insulin resistance enhances the inflammatory state due to the lack of insulin anti-inflammatory and vasodilatory effects. In addition, excess lipids that fail to be stored in the adipose tissue are deposited in other organs (such as liver, skeletal muscle, and blood vessels), leading to the expression of proinflammatory mediators, the differentiation of monocyte to macrophage, and the recruitment of M1 macrophages at a systemic level [10]. This chronic condition can establish a vicious circle characterized by increased central adiposity, intrahepatic fat accumulation, vascular inflammation, and impairment of endothelial function with cardiovascular sequelae of the central nervous system and various organs and tissues [11].

Recent evidence suggests that diet might be a key component of human life, playing a role as a determinant or suppressor of low-grade chronic inflammation, irrespectively of or in synergy with obesity [12]. The aim of this review is to explore current knowledge on the relation between dietary factors mediating inflammation, including the clinical evidence for food groups and dietary patterns exerting anti-inflammatory effects and the potential mechanisms potentially explaining the observed effects.

## 2. Postprandial-to-Chronic Intestinal-to-Systemic Inflammation

Early studies in this area showed that postprandial rise in oxidative stress and inflammatory biomarkers is a physiological process that occurs as a response to food ingestion: the mitochondrial emission of H2O2 following energy substrate processing leads to a reduction in cellular glutathione (GSH) content and increment in reactive oxygen species (ROS) generation, establishing a more oxidized redox state; as a result, transcription factors, such as NF-kB, are activated to induce the proinflammatory genes and the consequent upregulation of pro-inflammatory cytokines (such as various interleukins (ILs), tumor necrosis factor-alpha (TNF-alpha), and interferon-gamma (INF-gamma)), chemokines (such as MCP-1, IL-18, RANTES, MIP-2, CXCL1, CXCL10), and the expression of cyclooxygenase-2 (COX-2) and inducible nitric oxide synthase (iNOS) [13]. However, some differences between macronutrients can be observed: the isocaloric intake of lipids, proteins, and glucose has been shown to induce intracellular oxidative stress, with protein being the least effective and fats inducing the highest response [14]. Thus, different macronutrients may affect the inflammatory state in different manners, while improving knowledge of the gut microbiota and the interaction with the intestinal immune system lead to a deeper understanding not only of acute postprandial response but also of longer-term effects, such as whether the stimuli were sustained over time [15].

Macronutrient and, even more importantly, dietary patterns may lead to acute and chronic gut microbiota modifications, with changes in bacterial diversity and a shift from the abundance of *Bacteroides* to higher levels of *Firmicutes*, which can lead to the activation of the TLR signaling pathway (a variegated group of receptors recognize different pathogen-associated molecular pattern molecules) and increased permeability to endotoxins (such as lipopolysaccharides (LPS), the main component of Gram-negative bacterial cell wall). LPS may translocate to the systemic circulation through the absorption of dietary factors (transcellular) or through compromised enterocyte tight junctions (paracellular) [16]. The translocation of LPS to the circulation may activate pro-inflammatory pathways, including the NF-kB and mitogen-activated protein kinase (MAPK) pathways, leading to the increased production of inflammatory mediators [17]. When the alteration of the gut microbiota is sustained over time, the inflammatory state involves other tissues and systems, potentially leading to more systemic effects [18]. Importantly, a rise in the secretion of inflammatory mediators by macrophages and T cells, such as IL-6, triggers hepatocyte expression and the release of C-reactive protein (CRP), an acute-phase protein that is particularly useful for detecting acute tissue injury when expressed at higher concentrations (i.e., over 10.0 mg/L) but that lately has been considered as a marker of subclinical chronic inflammation when measuring small variations at minimum levels (i.e., high-sensitivity CRP) [19]. The CRP circulating level has been considered to be a reliable risk factor for cardiovascular disease mortality and has been associated with an increased risk of certain cancers and neurodegenerative disorders [20]. CRP exerts pro-inflammatory effects on several cell lines, including endothelial cells, leukocytes, and platelets, via the activation of various signaling pathways [21,22].

## 3. Major Food Groups, Dietary Patterns and Inflammation

**Figure 1:**
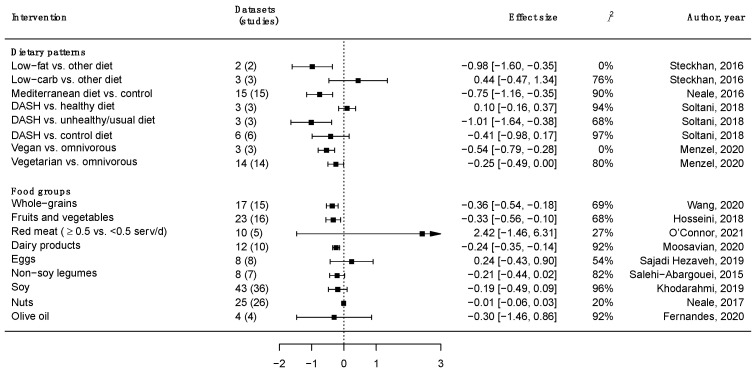
Summary results from meta-analyses on C-reactive protein levels changes in dietary intervention trials.

Among the many aforementioned inflammatory biomarkers, the most studied association with dietary factors by far is the potential effects on CRP levels [23]. A summary of current evidence from meta-analyses of randomized controlled trials (RCTs) on the effect of major dietary patterns and food groups on CRP levels is presented in Figure 1. Various food groups, such as whole grains [24], fruits, and vegetables [25], which are considered “healthy” due to their nutritional value (i.e., high content of vitamins, fiber, and phytochemicals), have been demonstrated to play a role in the human immune system by reducing the levels of inflammation (measured as serum CRP levels). Food groups rich in vegetable fats, such as olive oil [26] and nuts [26], as well as other plant-foods such as soy [27], legumes, and pulses [28], have been shown to cause a non-significant decrease in CRP levels. Among animal foods, dairy products [29] have shown a significant reduction effect on CRP levels, while others, such as red meat [30] and eggs [31], have been shown to produce null results.

While studies on individual foods are useful in order to better understand the potential role of single dietary factors in inflammation, studies on dietary patterns are better at describing the “real-world” scenario, where foods are consumed in combination and nutrients interact with each other with synergistic or antagonistic effects. Most existing evidence from meta-analyses of intervention studies shows that healthy dietary patterns, such as the Mediterranean diet [32] and the Dietary Approaches to Stop Hypertension trial (DASH) diet [33] may lead to a decrease in CRP level when compared to unhealthy options (Figure 1). The Mediterranean diet is a dietary pattern that has been widely studied over the last 50 years since the early observations of its beneficial effects on the cardiovascular risk of southern Italian individuals made in the 1960s [34]. While there are some practical differences between the dietary habits of populations living around the Mediterranean basin due to their different cultural heritage, history, and religious background, the main features of the Mediterranean diet have been identified scientifically and are described as follows: (i) a high consumption of plant-based foods, such as fruits, vegetables, whole-grain cereals and derivatives, nuts, and legumes; (ii) a moderate consumption of dairy, eggs, and fish as sources of protein; (iii) a low intake of meat and sweets; (iv) the daily consumption of olive oil; and (v) a moderate consumption of alcohol, in form of wine drunk during meals [35]. As a result, the Mediterranean diet can be considered a “high-fat diet”, with mono-unsaturated fatty acids (MUFAs) and poly-unsaturated fatty acids (PUFAs) being the most representative of the diet, accompanied by a high amount of fiber, antioxidant vitamins, and phytochemicals [36]. The DASH diet has been applied in the US as a healthy dietary model characterized by (i) an increased consumption of fruits and vegetables, whole grains, and nuts; (ii) consumption of low-fat dairy products, fish, and poultry as main sources of protein; (iii) a limited consumption of meat, sweets, and beverages containing sugars; and (iv) a recommended limited consumption of sodium [37]. In this way, this dietary model results in a low content of total fats (including cholesterol, trans-, and saturated fats) and rich content of minerals (magnesium, potassium, calcium) and fibers.

Evidence from studies focusing on certain aspects of dietary patterns has shown that the adoption of a vegetarian or even vegan diet would reduce CRP levels compared to an omnivorous diet [38]. Moreover, investigating the role of fats and carbohydrates revealed that a low-fat diet would have a stronger impact on CRP levels than a low-carb one, compared to other control diets [39]. This latest evidence suggests the potential role of macronutrients’ distribution in the diet, with a certain preferable positive effect of plant-based diets that are poor in animal fats. Additionally, results from observational studies support the hypothesis that a higher adherence to dietary patterns in line with the Mediterranean diet principles and, in general, a diet that is rich in plant-based foods (i.e., fruits, vegetables, whole-grain cereals, nuts, legumes, and vegetable oils) is associated with lower serum concentrations of CRP levels and a lower risk of elevated inflammatory biomarkers, while “Westernized” dietary patterns characterized by an excess intake of empty calories, processed meats, sodium, fats, and free sugar have been associated with higher levels of inflammatory biomarkers [40,41].

## 4. Dietary Fat Sources and Inflammation

Contrary to the idea that has arisen over the last few decades that dietary fats are substantially bad for human health, growing evidence suggests that foods rich in fats may exert different effects on health outcomes depending on the type of fat present and the characteristics of the so-called “food matrix”, defined as a physical form constituting a certain food in which specific constituents (i.e., nutrients) provide functionalities which are different from those exhibited by the same compounds when considered in isolation or a free state [42]. With regard to inflammation, the aforementioned results are quite inconsistent, showing null effects on levels of inflammatory markers following the consumption of red meat, a tendency toward a positive effect following the consumption of olive oil, and a significant positive effect (decrease in CRP levels) of dairy products. Summary results from postprandial studies on the effects of “high-fat meals” in healthy humans also showed that inflammatory biomarkers (i.e., CRP and TNF-alpha) were not consistently responsive [43]. Thus, accruing evidence thus suggests that not all fats univocally promote inflammation.

### 4.1. Saturated Fats

The evidence on the pro-inflammatory activity of saturated fatty acids is by far the most comprehensive in relation to high-fat meals and inflammatory biomarkers, with substantial contrasting results being found [44]. These findings may depend on the fact that most studies exploring the role of diets rich in saturated fatty acids do not distinguish between the types of molecules involved and hence lack specificity. Moreover, the detrimental effects on human health attributed to saturated fatty acids are currently under discussion, since recent meta-analyses have not provided convincing evidence of the association between higher contents of saturated fatty acids and increased risk of cardiometabolic outcomes [45]. There is an ongoing debate as to whether the chemical structures of saturated fatty acids may play an underrated and probably unmeasured role in the effects of various types of molecules on human health: for instance, long-chain fatty acids (palmitic acid (C16:0), with palm oil being the richest vegetable source, and stearic acid (C18:0), both found in meat and dairy products) may be responsible for pro-inflammatory activities, while medium-chain fatty acids (caprylic (8:0), capric (10:0), and lauric (12:0) acids), short-chain fatty acids (SCFA) (i.e., butyric (4:0), valeric (5:0), and caproic (6:0) acids), and branch-chain fatty acids (BCFAs) may potentially exert anti-inflammatory actions [46].

**Figure 2:**
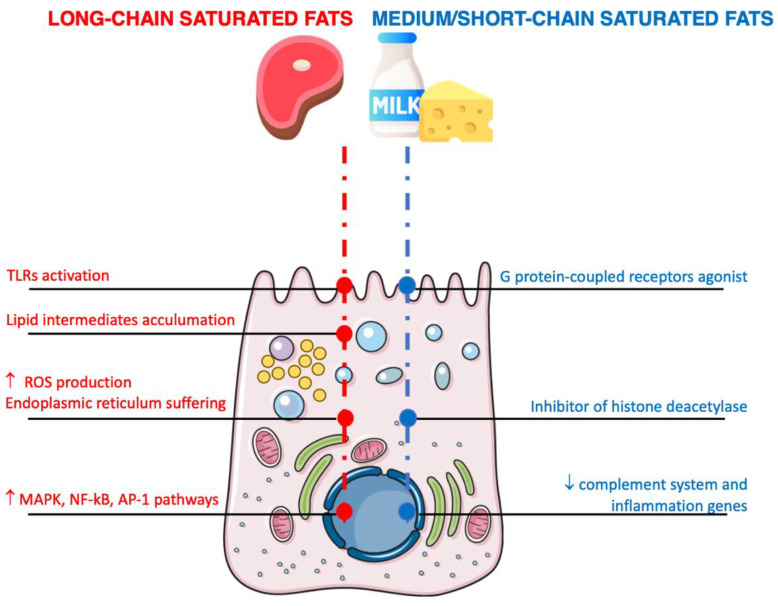
Mechanisms of action related to the effects of the inflammatory pathways of saturated fatty acids on intestinal cells. Arrows denote increment/increase or decrement/decrease.

Long-chain fatty acids may directly affect cell longevity due to production of ROS following oxidation in the mitochondria (Figure 2) [47]. Generally, free fatty acids entering adipocytes are converted to fatty acyl-coA and stored as triglycerides without significant oxidative processes occurring in the mitochondria [48]. However, excess levels of long-chain fatty acids may lead to the activation of reduced nicotinamide adenine dinucleotide phosphate (NADPH) oxidase and protein kinase C (PKC), which lead to the production of ROS, the accumulation of lipid intermediates (such as saturated phospholipids (i.e., lysophosphatidylcholine), diglycerides (i.e., diacylglycerol), and ceramides), and damage to the endoplasmic reticulum [49]. Excess concentrations of saturated hydrocarbon chains cause their accumulation in and subsequent destruction of the endoplasmic reticulum structure as well as the activation of stress sensors [50]. Long-chain fatty acids have been reported to activate a number of inflammatory pathways (including MAPK (such as p38, JNK, and extracellular-signal-regulated kinases), NF-kB, and activator protein (AP)-1), resulting in the increased expression of cytokines in macrophages, monocytes, and monocyte-derived dendritic cells [51]. As previously mentioned, the mechanisms underlying these potential effects may involve TLR4, the receptor able to specifically recognize LPS, and TLR2, another member of the TLR family that is able to recognize a broad range of ligands, including triacyl lipopeptide (LP), from Gram-positive bacterial cell walls [52]. Moreover, long-chain saturated fatty acids may directly upregulate the expression of TLRs both in intestinal cells and in circulating macrophages, thus activating the innate immune system and leading toward an increased local and peripheral inflammatory state [53]. The exact mechanisms through which long-chain fatty acids may activate the TLR family are still yet to be fully understood; the chemical structure of saturated fatty acids is similar to the components of bacterial endotoxins, but molecular simulations and theoretical studies have reported that saturated fatty acids are unlikely to act as actual ligands for such receptors [54]. In contrast, it has been hypothesized that saturated fatty acids may indirectly activate TLRs via recruitment into lipid rafts, which are submicroscopic cellular membrane microdomains composed of protein receptors and cytoskeletal elements (such as microtubules or actin microfilaments and the monomers or dimers that comprise them) enriched with cholesterol, sphingolipids, and glycolipids [55]. In fact, TLRs have been shown to be active when grouped into dimers: saturated fatty acids may facilitate this dimerization by affecting the physical composition of lipid rafts and the modeling of these complexes [56]. Another mechanism involves the modification of the gut microbiota taxonomy, dysbiosis, and increased intestinal permeability to LPS [57]. Whichever the predominant mechanism is (or perhaps the synergistic action of all of them), the resulting effect leads to an increase in LPS in the bloodstream with the direct stimulation of the immune system toward a pro-inflammatory response.

At the peripheral level, long-chain fatty acids have been reported to also act on hypothalamic neurons, which are designated to regulate food intake, energy expenditure, and ultimately body mass stability over time [58]. The hypothalamus responds to circulating adipostatic and satiety factors such as insulin, cholecystokinin, ghrelin, glucagon-like peptide-1 (GLP1), and leptin [59]. The consumption of high-fat diets has been associated with a higher expression of transforming growth factor-beta (TGF-beta) by hypothalamic astrocytes and the activation of NF-kB signaling in proopiomelanocortin (POMC) neurons, with both events leading to neural activity disfunction [60,61]. Inflammation may be triggered by long-chain fatty acids through the activation of TLR4 signaling in microglia, which leads to the release of cytokines; recruitment of monocytes; and the activation of intracellular serine/threonine kinases in hypothalamic neurons, which can interfere with key proteins of the leptin and insulin signaling systems, therefore disrupting adipostatic signals [62]. While the acute inflammatory response may be reversed if the stressor event is removed (i.e., a high-fat meal), chronic stimuli perpetuating inflammation may result in a lack of the inflammation resolution phase (when the body slowly restores the homeostatic physiological condition), the severe damage of hypothalamic neurons, and ultimately cell death through apoptosis [63]. The final overall result of the unresponsiveness of hypothalamic neurons to satiety and adipostatic factors causes a predisposition towards obesity.

Medium- and SCFA have different metabolic pathways in the human organism to those of long-chain fatty acids; these molecules can be absorbed in the gastrointestinal tract more efficiently and directly transported to the liver for rapid oxidation [64]. In contrast to long-chain fatty acids, medium- and SCFA have demonstrated a number of potential benefits concerning metabolic outcomes (Figure 2) [64]. With regard to inflammation, medium- and SCFA may be implicated in several immunomodulatory functions. Medium-chain fatty acids from dairy have been found to upregulate genes related to the citric acid cycle and oxidative phosphorylation (mostly concerning energy metabolism in the adipose tissue) and downregulate genes related to complement system and inflammation [65]. Among SCFA, butyric acid has been demonstrated to have a number of functions in the gut, acting as an inhibitor of histone deacetylase and an agonist of specific G protein-coupled receptors, demonstrating beneficial effects on metabolic outcomes (i.e., improved glucose metabolism, increased total energy expenditure, lower blood lipid levels, etc.) in preclinical studies [66]. However, these findings have been somewhat contrasting when replicated in humans, resulting in a modification of the intestinal microflora that may be affected by the weight status of participants (effective action was evident in lean individuals but not in obese ones) [67].

One particular group of saturated fats are BCFAs. These molecules are saturated fats substituted with one or more methyl branches on the carbon chain derived from rumen bacteria; they are structural lipid constituents of rumens’ bacterial membranes (and responsible for structural characteristics such as fluidity and permeability) that are subsequently absorbed and incorporated into milk fats [68]. Thus, the human intake of BCFAs mostly occurs through milk and dairy products, although they can also be found in fish and non-dairy fermented foods. There are more than 50 molecules identified among ruminant-derived BCFAs, but the most quantitatively abundant are 14–17 carbon atom chain-long iso- and anteiso-mono-methyl BCFAs [68]. Given their dietary sources, BCFAs may explain, at least in part, the health benefits associated with such food groups [69]. Concerning inflammation, preclinical laboratory studies on various cell lines have demonstrated the potential anti-inflammatory effects of BCFAs through the inhibition of the LPS-induced gene expression of classic pro-inflammatory transcription pathways (i.e., NF-kB and TLR-4) [70,71]. These findings have been replicated in some animal studies, in which BCFA-fed mice showed a lower incidence of necrotizing enterocolitis and enhanced expression of IL-10 [72]. Studies on humans are rather scarce, but emerging evidence has showed an inverse correlation between serum BCFAs (i.e., iso-15:0, iso-16:0, iso-17:0, and anteiso-15:0) and CRP levels [73].

### 4.2. Unsaturated Fatty Acids

**Figure 3:**
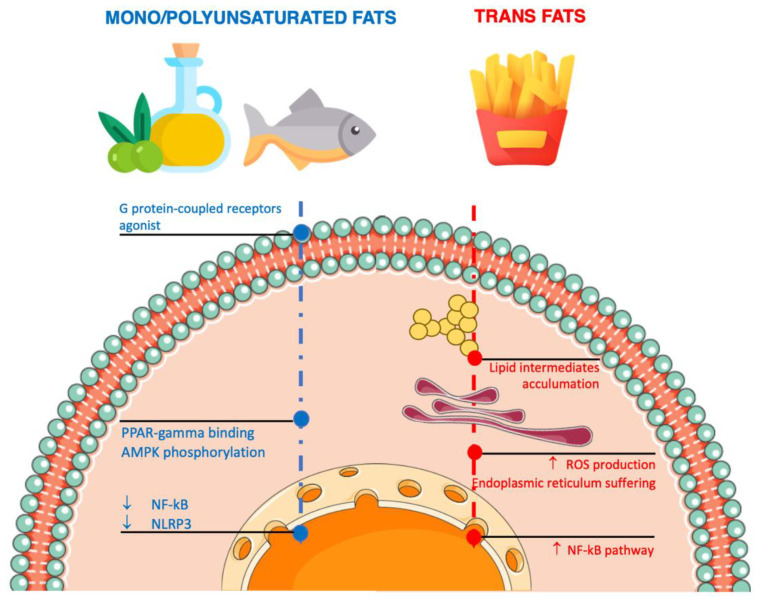
Mechanisms of action related to the inflammatory pathways of unsaturated fatty acids in adipocytes and macrophages. Arrows denote increment/increase or decrement/decrease.

Evidence regarding the role of MUFA and PUFA has evolved over time toward a stronger belief that this group of heterogeneous molecules may exert anti-inflammatory effects (Figure 3) [74]. One of the most common MUFAs, omega-9 oleic acid (18:1n-9, 9-octadecenoic acid) is present in high amounts in vegetable oils (especially olive oil) and certain nuts; in support of the aforementioned clinical findings on food sources rich in MUFA, several mechanisms have been proposed to explain such effects on inflammatory biomarkers. Preclinical mechanistic studies have shown that MUFA can exert anti-inflammatory actions by counteracting the effects of long-chain saturated fatty acids on hepatocytes, including limiting saturated fatty acid-induced lipotoxicity, reducing endoplasmic reticulum stress, decreasing ROS production, and inhibiting NF-kB transcription factors and nucleotide-binding oligomerization domain-like receptor pyrin domain-containing-3 (NLRP3) by binding peroxisome proliferator-activated receptor gamma (PPAR-gamma) and G-protein coupled surface receptor 120 (GPR120), as well as through AMPK (AMP-activated protein kinase) phosphorylation [75]. Other in vitro studies have shown the potential role of MUFA in inducing the expression of the adiponectin gene via PPARgamma activation, which would lead to a reduced production of IL-6 and TNF-alpha [76]. Moreover, other studies have shown that another potential mechanism of action by which MUFA could counteract inflammation is switching the polarization of M1 macrophages to M2 macrophages, which exert anti-inflammatory actions through the secretion of several anti-inflammatory factors such as MGL2, IL-10, TGFβ1, and MRC1 [77].

Concerning PUFA, earlier evidence from in vitro and in vivo studies has supported the hypothesis that n-3 and n-6 PUFA would have opposite effects on the immune system, with the former exerting an anti-inflammatory effect while the latter exerts a pro-inflammatory action [78]. The concerns regarding the n-6 PUFAs, such as linoleic acid (LA, 18:2n-6), contained in vegetable oils, nuts, seeds, meats, and eggs, are based on a number of potential mechanisms supported by a mechanistic and, probably, oversimplistic interpretation of the metabolic pathway of such molecules, leading to the promotion of conversion into arachidonic acid (AA; 20:4n-6, eicosatetraenoic acid) and the subsequent production of proinflammatory eicosanoids (such as PGE2 and leukotriene B4 (LTB4)), as well as the reduced conversion of alpha-linolenic acid (ALA, 18:3n-3, octadecatrienoic acid) into eicosapentaenoic acid (EPA, 20:5n-3) and/or docosahexaenoic acid (DHA, 22:6n-3) due to competition for the same enzymes (elongases and desaturases), resulting in a lower production of pro-resolving anti-inflammatory lipid mediators such as resolvins, docosatrienes, and protectins [79]. However, contrasting results have been reported when considering studies conducted on humans [80], which are further corroborated by evidence from clinical studies (randomized controlled trials) providing null evidence [81]; this evidence undermines the hypothesis of a pro-inflammatory action of dietary LA. These potential counterintuitive findings may depend on the fact that the biological effects of dietary LA on the immune system might be more complex than previously thought: in fact, a high variation in dietary LA has been demonstrated to lead to very small changes in AA (thus resulting in very limited pro-inflammatory stimulus) [82], accompanied by potential anti-inflammatory effects following the conversion of LA into nitrosylated LA and 13-hydroxyoctadecadienoic acid [83]. Thus, the fine equilibrium between such contrasting biochemical pathways has not been entirely and clearly elucidated yet. In contrast, ALA is an essential nutrient derived from vegetable sources (i.e., contained in certain seeds and nuts) that has long been considered a potential driver of dietary anti-inflammatory effects being the substrate for the reaction leading to the conversion of anti-inflammatory eicosanoids. However, a recent summary of evidence has revealed that its supplementation does not lead to any beneficial effects on most inflammatory biomarkers (such as TNF, IL-6, CRP, intracellular adhesion molecule-1 (ICAM-1), and vascular cell adhesion molecule-1 (VCAM-1)) [84]. Once again, it is important not to oversimplify the metabolic pathways under examination and underline the fact that ALA does not provide strong direct anti-inflammatory effects, with it rather serving as a substrate for the production of DHA and EPA, which are the real precursors of anti-inflammatory eicosanoids. Thus, as previously mentioned, DHA (and EPA) production is not only dependent on ALA intake, but also on enzyme saturation levels (also due to LA products or simply ALA supply). It is not surprising that a later summary of RCTs showed a stronger effect of DHA and EPA on inflammatory biomarkers (CRP concentrations in this case) [85]. Besides promoting the production of anti-inflammatory mediators, DHA and EPA may exert anti-inflammatory actions through other pathways similar to MUFA, such as the inhibition of NF-kB transcription factors by binding PPAR-gamma and certain G-protein-coupled surface receptors in adipocytes and macrophages, leading to a decreased production of inflammatory cytokines [86]. Moreover, DHA and EPA may reduce insulin resistance by activating PPAR-alpha and suppressing sterol regulatory binding protein-1c (SREBP-1c) [87]. However, it is important to underline that current evidence supporting this effect needs to be further investigated, as a discrepancy between preclinical studies has been pointed out, resulting in much larger concentrations of cellular EPA and DHA in murine immune cells with consequent stronger anti-inflammatory effects compared those seen in human ones [88]. Finally, n-3 PUFA may promote microbiome diversity in the large intestine and improve intestinal barrier function by sealing epithelial tight junctions [89].

Trans fatty acids are a particular type of unsaturated fatty acids with at least one non-conjugated carbon-carbon double bond in the trans configuration. Trans fats may naturally occur in food products derived from ruminant animals (accenate (18:1 trans-delta11)), but the major dietary sources in average diets (especially “Westernized” ones) are partially hydrogenated oils (i.e., elaidate (18:1 trans-delta9)), mostly of industrial origin (i.e., margarine) [90]. Consistent evidence from preclinical and human studies shows a pro-inflammatory response associated with a higher intake of industrial trans fats, while natural ones have been reported to exert null or mildly beneficial effects on inflammatory markers, although the evidence relating to this is rather contrasting [91]. Trans fatty acids may show pro-inflammatory action due to cellular toxicity effects (Figure 3); specifically, there is evidence of deleterious effects on endoplasmic reticulum stress, the accumulation of lipid intermediates (ceramides and diglyceride), ROS production, and oxidative stress [92]. The incorporation of trans fatty acids into cell membrane phospholipids may lead to the alteration of the membrane receptors with the subsequent modulation of nuclear receptors regulating gene transcription and signaling pathways related to inflammation (i.e., NF-kB) in monocyte/macrophage and adipocyte cells [93]. Laboratory studies on some cell lines have also suggested that exposure to trans fatty acids may stimulate autophagy, an adaptive response to stress characterized by degradation in the lysosome of organelles and other intracellular components in order to recoup energy substrates for cell survival [94]. However, the results of these studies are only preliminary and further research on humans is needed to confirm the findings. Another mechanism explaining the pro-inflammatory response to trans fats is direct action on the gut microbiota resulting in the local infiltration of colonic mucosa of CD68+ cells and vascular VCAM-1 and the systemic production of proinflammatory cytokines and markers for inflammation (IL-6 and CRP) via increased LPS absorption and the stimulation of macrophage activity [95].

## 5. Dietary Protein Sources and Inflammation

**Figure 4:**
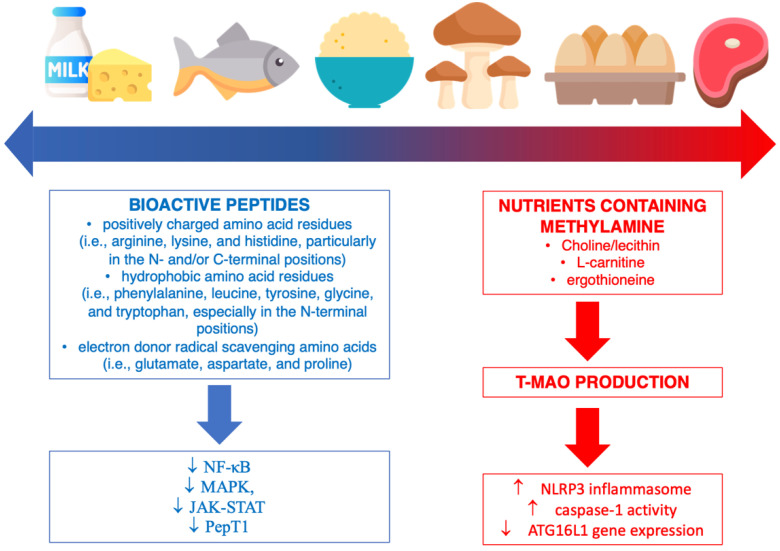
Mechanisms of action related to the inflammatory pathways of protein-rich foods. Arrows denote increment/increase or decrement/decrease.

Dietary proteins represent an important factor in supporting a healthy immune system, since amino acids play an essential role in the synthesis of the immune proteins, cytokines, antibodies, and enzymes involved in the inflammatory response as well as the regulation of adaptive immunity and innate immune cells (B and T cells, NK cells, and macrophages) [96]. The potential anti-inflammatory effects observed following the consumption of certain protein sources, such as fish/seafood and dairy products, has been attributed, at least in part, to bioactive peptides (Figure 4) [97,98]. Although dairy products are the richest sources of bioactive peptides, they are also obtained from other dietary sources, including fish (such as tuna, sardine, anchovy, herring, and salmon), eggs, and meat products (i.e., bovine muscle and blood), as well as vegetable sources, such as wheat, maize, soy, rice, amaranth, sorghum, and mushrooms [99]. These compounds are derived from various types of protein sources and may be produced following protein hydrolysis during digestion (from digestive or microbial enzymes) or food processing (i.e., the transformation of milk into other dairy products) [98]. Bioactive peptides consist of 2–20 amino acids characterized by some features of their chemical structure that may provide anti-inflammatory effects, such as (i) the presence of positively charged amino acid residues (i.e., arginine, lysine, and histidine, particularly in the N- and/or C-terminal positions) that have been demonstrated to have anti-inflammatory activity against LPS-specific-stimulated inflammatory responses and to activate chemokine receptors; (ii) the presence of hydrophobic amino acid residues (i.e., phenylalanine, leucine, tyrosine, glycine, and tryptophan, especially in the N-terminal positions), which have been shown to interact with cell membranes and potentially disrupt inflammatory pathway cascades (i.e., binding Ca^2+^ and disrupting NF-κB signaling and cytokine production); (iii) the presence of electron-donor radical-scavenging amino acids (i.e., glutamate, aspartate, and proline) able to exert antioxidant activity and protect against ROS-induced inflammation [100]. Furthermore, it has been noted that bioactive peptides exhibit better anti-inflammatory properties when they have a short chain, since they are more likely to cross the intestinal barrier intact and be more rapidly absorbed [101]. From a mechanistic point of view, the main pathways demonstrated in both in vitro and in vivo models to be affected by bioactive peptides are the NF-κB, MAPK, Janus kinase-signal transducer and activator of transcription (JAK-STAT), and peptide transporter 1 (PepT1) pathways [102]. Moreover, bioactive peptides and hydrolysates may stimulate the immune system by increasing the killing activity of NK cells, increasing the phagocytic activity of macrophages, and increasing lymphocyte populations as well as antibody and cytokine production [102]. Additional potential effects demonstrated for some bioactive peptides that could indirectly affect the inflammatory state include antioxidant activity (i.e., inhibiting lipid oxidation, metal chelating, and reducing activities), anti-hypertensive activity (i.e., angiotensin-1-converting enzyme inhibitory activity), antimicrobial activity (i.e., direct activity against Gram-positive or Gram-negative pathogenic bacteria), and activity regulating gut–microbiota imbalances (i.e., normalizing the population of *Bacteroidetes* and *Firmicutes* in the colon) [103]. The progress made in proteomics techniques is helping to produce a growing amount of evidence on this subject, but further studies are needed to better elucidate the potential effects of bioactive peptides in in vivo settings.

Several protein sources have also been found to be related to pro-inflammatory responses due to their content of nutrients containing methylamine (Figure 4). Among these compounds, choline/lecithin (contained in fish, eggs, and some dairy products), L-carnitine (contained in red meat), and ergothioneine (contained in mushrooms, some meat products, and several types of beans) are transformed by the gut microbiota into trimethylamine (TMA), then subsequently absorbed, transported to the liver, and oxidized to TMA N-oxide (TMAO) by flavin-dependent monooxygenase (FMO) isoforms 1 and 3 [104]. The importance of exploring the deleterious effects of TMAO production on human health is related to the evidence regarding the increased risk of non-communicable diseases (mainly cardiovascular and neurodegenerative diseases) due to the rise in inflammatory state as the main underlying mechanism [105]. Several cell lines have been tested with TMAO, producing a dose- and time-dependent inflammatory response via the activation of the NLRP3 inflammasome, caspase-1 activity, and the inhibition of the expression of the ATG16L1 gene (coding for autophagy-related 16-like 1 protein), which is responsible for autophagy and immune system regulation through innate and adaptive immunity activation and pro-inflammatory cytokine production [106]. It is important to underline that, irrespective of the dietary intake, TMAO production seems to strictly depend on the gut microbiota profile (i.e., the Firmicutes/Bacteroidetes ratio may predict TMAO concentration in plasma) [107]. Thus, it is crucial for future studies to take into consideration the individual composition of the microbiome when exploring the relation between TMAO and inflammatory response.

## 6. Dietary Carbohydrate, Fiber and Inflammation

Carbohydrates are essential for the healthy development of the immune system [108]. There is growing evidence that carbohydrates may play a role in foreign and self-antigen recognition by T-cells and act as antigens modulating adaptive immune responses [109]. Moreover, the complex carbohydrates expressed on cellular glycoproteins and glycolipids are recognized by cell adhesion molecules (i.e., selectins, galectins, and other lectins) involved in leukocyte recruitment to sites of inflammation and activation of immunogenicity against external stressors [110]. However, different types of carbohydrates seem to exert different effects on the inflammatory response.

### 6.1. Fiber

**Figure 5:**
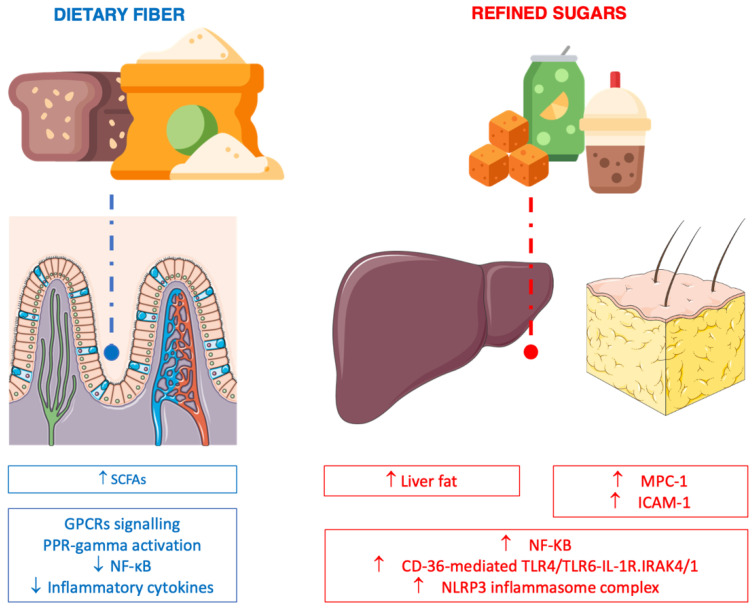
Mechanisms of action related to the inflammatory pathways of dietary carbohydrates. Arrows denote increment/increase or decrement/decrease.

Dietary fiber supplementation has been shown to decrease CRP levels in diabetic individuals [111], while another recent meta-analysis on the effects of resistant starch interventions on circulating inflammatory biomarkers showed a substantial reduction in the IL-6 and TNF-alpha levels, although no significant changes were found in CRP concentrations [112]. Additionally, a meta-analysis on low-glycemic-index diets (rich in fiber) reported a significant decrease in IL-6 in patients with type 2 diabetes compared to those with a higher-GI diet [113]. The potential anti-inflammatory action of dietary fiber may rely on a variety of mechanisms (Figure 5). The nutrient and non-nutrient composition of the food matrix of dietary sources of fiber seems to determine the anti-inflammatory role of such foods. Specifically, a concomitant intake of a variety of polyphenols (i.e., monomeric flavonoids) and carbohydrates (i.e., neutral sugars in cereal grain walls or ionic polysaccharides in legumes) has been hypothesized to lead to, to a varying extent, the modulation of the immune system through the downregulation of the activation of the NF-κB pathway and a reduction in cytokine production [114]. Moreover, SCFAs are produced by certain gut microbes in response to the ingestion of foods rich in fiber [115]. In fact, non-digestible carbohydrates (such as cellulose, hemicelluloses, pectin, inulin, fructose oligosaccharides, galactose oligosaccharides, polydextrose, and lignin) are processed by the gut microbiota through microbial carbohydrate-degrading enzymes (such as glycoside hydrolases, polysaccharide lyases, carbohydrate esterases, cellobiohydrolase, beta-glycosidase, arabinofuranosidase, and beta-fructofuranosidases) and turned into metabolites (acetate, propionate, butyrate) and gases [115]. Besides being used as substrates for energy production in the mitochondria of colonocytes (butyrate) and hepatocytes (propionate), SCFAs may modulate the immune system after being converted into acetyl-CoA to promote acetylation and activate signaling pathways through G protein-coupled receptors (GPCRs), such as free fatty acid (FFA) receptors type 2 and 3 (FFA2 and FFA3 receptors), and, in the nucleus, promote gene transcription via the inhibition of histone deacetylases (HDACs). The following activation of PPAR-gamma and inhibition of NF-kB activity result in a substantial anti-inflammatory activity, such as the inhibition of the production of inflammatory cytokines, TNF-α, MCP-1, and IL-6 via the activation of GPR41 in macrophages [116]. Moreover, SCFAs are important for gut microbiota diversity and gut integrity, which play a role in the inflammatory responses of the host organism. In fact, certain bacterial species may promote a “leaky gut” due to a loss of intestinal barrier function and lead to the passage of metabolites associated with microbes, such as LPS, secondary bile acids, and TMAO, into the bloodstream after transformation by gut microbes, as well as CRP production in response to an increase in certain bacterial species, all effectively promoting an inflammatory response [117]. SCFAs can also play a crucial role in the expression of tight junction proteins (through the upregulation of genes encoding tight-junction proteins) in order to regulate paracellular permeability and solute transport through the channels between intestinal cells [118].

### 6.2. Processed (Refined) Carbohydrate

Processed (refined) carbohydrates are foods that are heavily processed in order to remove certain components (i.e., bran, germ, and endosperm), resulting in a loss of nutrients they would normally carry [119]. Processed carbohydrates also include free added sugars, defined as all monosaccharides and disaccharides added to foods (different from free sugars naturally present in honey, syrups, and fruit juices) [120]. There is also evidence that free added sugars [121,122] may affect the inflammatory status, although a systematic assessment of studies related to this matter is still not available. Major global contributors to free added sugars have been demonstrated to be sugar-sweetened beverages [123], which have been shown to exert pro-inflammatory effects in observational studies [124,125,126,127,128,129,130] and a few older RCTs [131,132], although this has not been unequivocally demonstrated in an acute setting [133]. Interestingly, no evidence of pro-inflammatory effects has been found in controlled interventions with naturally occurring free sugars (i.e., fructose or high-fructose corn syrup) compared to other dietary sugars [134].

The most challenging question when studying processed carbohydrates is whether a direct mechanism of action toward a pro-inflammatory response (chronically sustained) exists aside from the well-known effects on liver and fat tissue, led by an overload of sugars (especially fructose) turning into fat and, subsequently, triggering inflammation (Figure 5) [135]. Nonetheless, other mechanisms have been hypothesized to drive a pro-inflammatory response irrespective of fat accumulation. For instance, several studies have pointed out the possible role of a chronic high intake of free added sugars (i.e., from sugar-sweetened beverages) and free sugars (i.e., fructose) in the alteration of the intestinal flora, dysbiosis of the gut microbiota, and increased permeability of the gut barrier [136]. Moreover, the inclusion of excessive fructose into cells and tissues leads to direct immune system activation through NF-kB signaling and (CD36)-mediated TLR4/TLR6-IL-1R–associated kinase 4/1 (IRAK4/1) signaling, thereby leading to the activation of the NLR family NLRP3 inflammasome complex and resulting in an increase in macrophage infiltration into adipocytes via MPC-1 and ICAM-1 induction [137]. Moreover, another hypothesized mechanism involves the catalytic effects of free sugars on advanced glycation endproduct (AGE) formation. AGEs are toxic compounds derived from proteins or lipids following a non-enzymatic glycoxidation reaction with sugars, resulting in structurally and functionally compromised products [138]. Growing evidence suggests that a high intake of dietary free sugars can represent a substantial source of endogenous AGEs, which have been suggested to potentially activate some NLRP3 receptors (such as receptor for AGEs (RAGE) and Galectin-3) acting on inflammasome assembly (i.e., through the activation of the NFkB pathway as well as still-unknown pathways) [139].

## 7. Dietary Polyphenols and Inflammation

Over the last few decades, a significant body of research has focused on the health effects of bioactive components of plant-based foods, such as polyphenols [140]. Polyphenols are a heterogeneous class of plant metabolites mostly known for their antioxidant properties [141]. There is evidence that a higher intake of some groups of dietary polyphenols (i.e., flavonoids) may reduce the risk of major non-communicable diseases, including cardio-metabolic diseases [142,143,144,145], neurodegenerative diseases [146], and certain cancers [147,148]. However, under certain conditions related to metal-reducing potential, chelating behavior, pH, and solubility characteristics, polyphenols may exert dual antioxidant and pro-oxidant activities [149]. More recently, the role of polyphenols in preventing non-communicable diseases featuring systemic chronic inflammation has been noted in research into their potential anti-inflammatory properties, with them either acting as anti-inflammatory agents through strengthening antioxidant activities or displaying regulatory properties in various pathways of inflammation [149]. In particular, oxidative imbalance causes the upregulation of pro-inflammatory cytokines (such as TNF-alpha and IL-6) and inflammatory molecules (such as VCAM-1, ICAM-1, and NF-κB). Likewise, inflammatory processes induce oxidative stress and reduce cellular antioxidant capacity through the upregulation of certain cell enzymes (such as NOX and XO), and, as a consequence, increased ROS release [17,150].

There is also evidence that dietary polyphenols may modulate inflammation by targeting innate and adaptive immunity systems [151]. In particular, specific phenolic molecules have been demonstrated to affect the cellular component of the innate system through various pathways. These mechanisms include the modulation of dendritic cells differentiation and maturation [152], as well as the regulation of the lytic activity of natural killer cells [153]. Additionally, polyphenols may mediate inflammation by affecting macrophage polarization and re-polarization, from pro-inflammatory (M1) to anti-inflammatory (M2) macrophages [154]. Although data on this topic are limited, polyphenols also contribute to the regulation of the adaptive immune system by affecting the function of B cells and the differentiation of T cells [151].

Another mechanism through which polyphenols regulate inflammation involves the gut microbiota. Once ingested, dietary polyphenols undergo a process of absorption and metabolism [155]. Though mainly unabsorbed in the upper gastrointestinal tract, polyphenols reach the colon, where they are metabolized by pivotal fecal microbiota in a wide range of well absorbable and bioactive phenolic metabolites [156]. Considerably, while the process of their metabolism depends on the host gut microbiota composition and other dietary components acting as co-factors [157], the evidence suggests that polyphenols themselves may alter the gut microbiota composition and function by influencing the ratio of beneficial and harmful bacteria taxes [158]. Indeed, dietary polyphenols have been shown to mitigate inflammation by increasing the fecal abundance of *Bifidobacteria* and *Lactobacilli* while inhibiting LPS producers such as *E. coli* [158]. Furthermore, a decreased release of LPS prevents a cascade of events, including the activation of immune cells through TLR4 and the downstream activation of NF-κB [159]. In this manner, polyphenols may decrease inflammation, which is attributable to lower LPS release [160]. Finally, several studies have shown that dietary polyphenols possibly exert effects on inflammation by preventing the disruption of the gut barrier via modulating the expression of tight junction proteins—namely, claudins and occludins [161,162].

## 8. Controversial Interpretation of Current Evidence

Although most mechanisms described seem to provide a relatively strong rationale for a direct effect of dietary factors on inflammation, there is still a vast area of research that needs to be explored in light of the following considerations.

Dietary intervention studies should further investigate the substitution effect of a certain food or nutrient, since studies currently taking into account such aspects have obtained contrasting results that are hard to interpret. While this approach has been widely investigated in relation to other outcomes, such as blood lipid levels, revealing beneficial effects of the consumption of whole grains and unsaturated fats over trans-fatty acids and refined sugars, as well as substituting saturated fats with PUFAs, data on inflammatory markers are still emerging [163]. For instance, an early RCT showed that, regardless of the intervention design (low fat/carbohydrate/protein diet), weight loss led to a decrease in inflammatory biomarkers, suggesting that a reduction in adipose tissue may represent the strongest stimulus affecting the inflammatory state [164,165]. However, other studies investigating the role of specific types of macronutrients showed a better inflammatory profile by substituting carbohydrates and saturated fats with unsaturated fats in individuals with metabolic syndrome [166,167,168]. Further research should also focus on the substitution effect of different types of macronutrients in order to better understand the mechanisms underlying specific groups of molecules within the context of the overall diet.

Another limitation is the fact that intervention studies on inflammatory biomarkers need to better assess baseline levels and better adjust for other factors that may play a role in chronic inflammation. There is substantial evidence of the effect of smoking [169], physical activity [170], and weight status [171] on inflammatory biomarkers. Additionally, pre-existing conditions (i.e., non-communicable diseases) may lead to an activation of the immune system and an establishment of a pro-inflammatory state. Besides the most obvious specific pathways related to the pathophysiological changes associated with the disease, there is emerging evidence of a substantial interaction between dietary choices, the alteration of the gut microbiota, the establishment of systemic inflammation, and the occurrence of disease, which may also occur the other way around [172]. Additionally, genetics has been shown to play a pivotal role in the inflammatory response to dietary exposure. For instance, it has been reported that up to about 60% of the heritability of lipid response to a high-fat diet may depend on genetic heritage, with BMI, sex, and age being the major effect modifiers [173]. Overall, while only hinted at in the context of this review, there is evidence that several variables play a role in the effects of diet on inflammation, which needs to be further addressed. Regardless of the magnitude of influence of macronutrient intake on inflammation pathways, the overall effect on the risk of non-communicable diseases is likely to also be influenced by the health status of a person, resulting in an exacerbated inflammatory response in obese individuals and those with cardio-metabolic conditions in general [174]. Although general recommendations may lay the groundwork for nutritional guidelines, the path toward personalized nutrition will be an increasingly desirable area of research in the near future.

Finally, it is also important to question the use of inflammatory biomarkers for assessing the level of low-grade chronic inflammation. Most existing clinical studies used CRP levels as a surrogate for chronic inflammation, since small variations in CRP are established when the liver is involved and inflammation is no longer localized in the gut and reaches systemic levels. However, unsolved questions regarding the early stages of the inflammatory process still remain. Some studies have shown that monocyte to macrophage differentiation is related to important changes in the lipid-related transcriptome [175]; specifically, certain plasminogens (i.e., phosphatidylethanolamine), which are structural components of the cell membrane that are primarily synthesized in the liver and highly influenced by MUFA and PUFA, may be considered potential biomarkers of cell activation and may protect the liver from steatosis and steatohepatitis [176]. There is no general consensus as to which biomarker of inflammation best characterizes low-grade inflammation related to food intake. It is understandable that the use of a combination of several markers following defined changes (i.e., diet over the other factors potentially influencing inflammation) would probably be a better approach to investigate the inflammatory state of a person [177].

## 9. Conclusions

In conclusion, the role of diet in inflammation is currently under investigation and evidence regarding the effects of certain foods on the immune system has emerged. Several mechanisms have been identified, revealing an interpretation of findings more complex than previously hypothesized, especially when comparing preclinical mechanistic studies to clinical interventions in humans. Over the past few years, some concepts have been considered in an oversimplistic manner and need to be refuted or at least considered in the light of a more complete interpretation. However, there is evidence that dietary factors may directly affect the immune system and play a role in systemic chronic inflammation. Although obesity-related inflammation may represent a crucial determinant for chronic subclinical inflammation, the modulation of the immune system through the diet may represent a crucial strategy to reduce the risk of non-communicable diseases. Preferential consumption of plant-derived foods and dairy products is likely to exert anti-inflammatory effects in humans. The contrasting results obtained for animal products, such as red meat and eggs, suggest that moderate consumption is physiologically beneficial for omnivorous beings such as humans, while the potential pro-inflammatory effects of such foods may be suppressed by the consumption of fiber-rich foods.

## Abbreviations

Toll-like receptors (TLRs); tumor necrosis factor receptors (TNFRs); interleukin-1 receptor (IL-1R); high activation of the nuclear factor-kB (NF-kB); glutathione (GSH); reactive oxygen species (ROS); various interleukins (ILs); tumor necrosis factor-alpha (TNF-alpha); and interferon-gamma (INF-gamma); cyclooxygenase-2 (COX-2); inducible nitric oxide synthase (iNOS); lipopolysaccharides (LPS); mitogen-activated protein kinase (MAPK); C-reactive protein (CRP); Dietary Approaches to Stop Hypertension trial (DASH); mono-unsaturated fatty acids (MUFA); poly-unsaturated fatty acids (PUFA); short-chain fatty acids (SCFA); branch-chained fatty acids (BCFAs); reduced nicotinamide adenine dinucleotide phosphate (NADPH); protein kinase C (PKC); activator protein (AP)-1; triacyl lipopeptide (LP);glucagon-like peptide-1 (GLP1); transforming growth factor-beta (TGF-beta); proopiomelanocortin (POMC); nucleotide-binding oligomerization domain-like receptor pyrin domain-containing-3 (NLRP3); peroxisome proliferator-activated receptor gamma (PPAR-gamma); G-protein coupled surface receptor 120 (GPR120); AMPK (AMP-activated protein kinase); alpha-linolenic acid (ALA); eicosapentaenoic acid (EPA); docosahexaenoic acid (DHA); intracellular adhesion molecule-1 (ICAM-1); vascular cell adhesion molecule-1 (VCAM-1); sterol regulatory binding protein-1c (SREBP-1c); Janus kinase-signal transducer and activator of transcription (JAK-STAT); and peptide transporter 1 (PepT1); trimethylamine (TMA); Trimethylamine N-oxide (TMAO); G protein-coupled receptors (GPCRs); free fatty acid (FFA); histone deacetylases (HDACs); glycemic index (GI); advanced glycation endproducts (AGEs); receptor for AGEs (RAGE).
